# Proceedings of the Annual Convention of the Connecticut Society

**Published:** 1846-09

**Authors:** 


*Proceedings of the Annual Convention of the Connecticut Society,* May, 1846. together with a list of members and the Annual Address.

OUR thanks are due to Dr. Russell, secretary of the above named medical society, for a copy of the Proceedings of its last annual convention. There are connected with this society, as shown by the published list, three hundred and fifty-eight members. It has been in existence, for more than half a century, and during which time, has made many valuable contributions to the literature of medicine.

The annual address delivered at the last meeting, by Theodore Stile, M. D., was on typhous fever. It is an able paper, exhibiting much close and critical observation, and a perusal of it, can hardly fail to be productive of profit.—*Balt. Ed.*

